# Acceptance and Online Interpretation of “Gender-Neutral Pronouns”: Performance Asymmetry by Chinese English as a Foreign Language Learners

**DOI:** 10.3389/fpsyg.2021.765777

**Published:** 2022-03-02

**Authors:** Zheng Ma, Shiyu Wu, Shiying Xu

**Affiliations:** ^1^School of Foreign Languages, Shanghai University, Shanghai, China; ^2^School of Foreign Languages, Shanghai Jiao Tong University, Shanghai, China

**Keywords:** singular *they*, self-paced reading, language learning, pronoun systems, cross-linguistic influence, acceptability judgment test

## Abstract

The present study (*N* = 109) set out to examine the role of cross-linguistic differences as a source of potential difficulty in the acceptance and online interpretation of the English singular *they* by Chinese English as a Foreign Language (EFL) learners across two levels of second-language proficiency. Experiment 1 operationalized performance through an untimed acceptability judgment test and Experiment 2 through a self-paced reading task. Statistical analyses yielded an asymmetric pattern of results. Experiment 1 indicated that unlike native English speakers who generally accepted the singular *they* with all antecedent types, two Chinese EFL groups consisting of English majors (higher level) and non-English majors (lower level) both rated it as the least acceptable pronoun regardless of their proficiency level. In contrast, Experiment 2 demonstrated that like native English speakers, both Chinese EFL groups were not disrupted in their reading by the use of the singular *they* most of the time, although its online interpretation was modulated by L2 proficiency levels of the participants. While the English majors were not affected by the use of the singular *they*, the non-English majors spent a significantly longer time reading the latter region of the sentences where the singular *they* was used. In short, the results of the two experiments seem to indicate that under no time constraint, L2 speakers showed a heightened degree of grammar sensitivity, whereas when there was a time constraint, their grammatical sensitivity was reduced by a greater need to focus on meaning. The difficulty for Chinese EFL learners to acquire the singular *they* may be located at the restructuring of their existing knowledge of the plural feature of *they* [−PLURAL] in their mental lexicon and the adaptation to the sociocultural norms of the target language. The pedagogical implications of the findings are discussed.

## Introduction

It has been well documented that cross-linguistic factors exert a significant influence on the learning of a target feature in a second language (L2; Odlin, 2003; Jarvis and Pavlenko, 2008). Although cross-linguistic similarities do not always mean ease for L2 acquisition and differences do not necessarily create difficulties, ample research has shown that certain differences between the native and target languages may pose significant challenges (Shatz, 2017; Liu et al., 2020). While congruence between L1 and L2 of learners can lead to positive L1 transfer, incongruence may lead to negative transfer, resulting in learner difficulties (Ding and Reynolds, 2019).

One area where languages may differ is the use of pronouns. For example, while in English, *they* is the only third-person plural form, there are two third-person plural forms in Mandarin. They are homophones derived by adding the plural marker 们 [*mén*] to the two separate singular third-person pronouns, namely, 他 [*tā*—*he*] and 她 [*tā*—*she*] (Qiu, 2013). What is especially notable is that the English plural pronoun, *they*, has emerged as a gender-neutral pronoun and has become the pronoun of choice of English speakers when referring to a singular non-gendered antecedent (LaScotte, 2021). In Mandarin, however, no counterpart pronoun can be found. Given these cross-linguistic differences, a question arises: Can Chinese learners of English as a foreign language (EFL) attain a native-like performance level in their use of the singular *they*? To answer this question, the present study aims to identify the possible sources of difficulty for Chinese EFL learners in acquiring the singular *they* by specifically focusing on the influence of both internal and external L1-related factors.

### Rise of the Singular *They* in English

In linguistics, a pronoun is a word used as a substitute for a noun or a noun phrase. In prescriptive grammar, the gender and number of a pronoun must agree with those of its antecedent. Consider the following:

(1) a. The patient should be told at the outset how much *they* will be required to pay.
b. But a journalist should not be forced to reveal *their* sources.
c. This is my friend, Jay. I met them at work. *They* are a talented artist.

In keeping with this gender/number agreement rule, the three sentences in (1) are grammatically unacceptable given that the pronouns used as *they* and its inflected or derivative forms clearly violate the number agreement. However, *they*, used as a gender-neutral singular pronoun (hereafter singular *they*), has become ubiquitous in English (Balhorn, 2004). For example, *they* in (1c) was named Word of the Year for 2015 by the American Dialect Society and for 2019 by Merriam-Webster. In 2019, the American Dialect Society also selected it as Word of the Decade for the 2010s (see Arnold et al., 2021).

It should be noted that the sentences in (1) demonstrate two different uses of the singular *they*, depending on “whether the antecedent is a generic or hypothetical person (1a and 1b) or whether *they* refers to a named or otherwise definite person (1c)” (Bradley, 2020, p. 2). The present study, however, focuses only on the first use given its much wider acceptance and the innovative and still ongoing nature of the second use (see Bjorkman, 2017; Bradley, 2020).

Researchers have also conducted empirical studies to examine whether speakers have difficulty in the acquisition and comprehension of the singular *they*. Through corpus analysis, Paterson (2011) found that English-speaking children received input on the singular *they* when acquiring personal pronoun paradigms. *Via* a comparative frequency analysis in connection with the input and output of different pronouns in the CHILDES database, Paterson concluded that the singular *they* is already distinct from the plural *they*, and that it may be acquired as a separate form in the mental lexicon. Researchers have also conducted empirical studies to examine whether the singular *they* causes confusion and processing costs during reading. A study consisting of two self-paced reading (SPR) experiments was conducted by Foertsch and Gernsbacher (1997). In their first experiment, native English undergraduate students were asked to read a group of three-clause sentences. Their reading times for *he*, *she*, and the singular *they* were recorded and compared under different types of antecedents that were either gender-neutral, stereotypically feminine, stereotypically masculine, or indefinite pronouns. The results indicated that the singular *they* was read either faster than *he* and *she* when the antecedents were indefinite pronouns or with equal facility when the pronouns matched the stereotypical gender of the antecedent (e.g., *he* and *a truck driver*). In their second experiment, modifiers were added to all the antecedents in the first clause so that all the indefinite antecedents became referential ones. This change gave the reader the impression that each sentence was about a specific person whose gender was presumably known. It was found that clauses containing the singular *they* were not read as quickly as those containing a gendered pronoun that matched the stereotypical gender of the antecedent but were still read faster than the pronouns that did not match the stereotypical gender of the antecedent. Based on these findings, Foertsch and Gernsbacher concluded that the singular *they* incurred no costs in the reading of native English speakers and that it was a cognitively efficient substitute for the generic *he*.

Since Foertsch and Gernsbacher (1997) and Paterson (2011), further studies have emerged (Sanford and Filik, 2007; Bjorkman, 2017; Bradley, 2020; Konnelly and Cowper, 2020; Arnold et al., 2021). Although there are minor variations in the results (e.g., Sanford and Filik, 2007), these studies generally support the acceptance and increasingly wider use of the singular *they* in English. In addition, researchers have noticed a more recent change occurring in the use of the singular *they*: it is even used with an antecedent that is singular, definite, and specific, “referring to an individual whose binary gender is known to both speaker and hearer” (Bjorkman, 2017, p. 2), as illustrated in (1c) above.

### L2 Acquisition of the Singular *They*

A question of both theoretical and practical importance in second-language acquisition studies then arises as to whether non-native learners of English can develop a native-like performance level in accepting the singular *they*. As discussed above, the singular *they* is unique in that it is seemingly ungrammatical for those following the conventions of prescriptive grammar, and its use is not only a linguistic one, but also a sociocultural or pragmatic one. However, to date, only a limited number of empirical studies have investigated this phenomenon. Stormbom (2020) employed an online writing task as an elicitation tool to investigate variations among L2 speakers from eight European L1 backgrounds in the distribution of epicene pronouns and found that the use of the singular *they* by L2 speakers was heavily influenced by cross-linguistic factors. While some L1 groups conceptualized the singular *they* as a separate pronoun from the plural *they*—especially in antecedents with notional plurality—some other L1 groups only accepted the generic *he*. This finding was further confirmed by LaScotte (2021). *Via* a small-scale online writing survey, LaScotte found that some international English-as-a-second-language (ESL) students used the singular *they* in their writing, but a large number of them preferred to use the generic *he*, and a very large number misidentified the antecedent of the singular *they* in the given context. Very little research has been conducted to investigate the acceptance and processing of the singular *they* in an EFL context where the linguistic input containing the singular *they* is much more limited than in an ESL context, such as the international students in English-speaking countries in the study by LaScotte. The only known study that investigated the online processing of the singular *they* by L2 speakers is a recent one by Speyer and Schleef (2019) which dealt with German speakers learning English as an additional language. By adopting a related research paradigm with similar reading materials, the researchers attempted to replicate the findings with L2 speakers obtained in the original experiment of Foertsch and Gernsbacher (1997). They reported an encouraging picture of the way in which the singular *they* is acquired in L2. While the situations for the stereotyped antecedents were complex (i.e., the singular *they* presented challenges for the intermediate proficiency group), the advanced L2 speakers did develop native-like performance in the acceptance of the singular *they*, and their performances were “matching those of native speakers in almost all respects” (Speyer and Schleef, 2019, p. 797).

However, some important questions remain. It is assumed that the difficulty for an EFL/ESL learner to acquire the singular *they* may stem mainly from two major types of sources: an internal one and an external one. Regarding the first, the most significant internal source of difficulty is likely to be attributable to cross-linguistic influence. It has been well documented that EFL/ESL learners are heavily influenced by their first language (L1) knowledge base and prior experience of learning and using the pronoun system of their L1 when learning the target pronoun systems (see Antón-Méndez, 2010; Dong and Jia, 2011; Dong et al., 2014; Arnold et al., 2018). As for the second type, the major external source of difficulty in acquiring the singular *they* concerns the limited language input and exposure that a learner can receive. The singular *they* is not typically taught in textbooks or in classroom settings (LaScotte, 2021). As a result, classroom learners of English do not generally receive sufficient input of the singular *they* that is needed to develop a robust representation of this form in their grammar (Speyer and Schleef, 2019).

While previous studies have examined the effects of these internal and external sources of difficulty in English pronoun learning separately, to the best of our knowledge, no study has been conducted on the effects of the two sources simultaneously. Chinese EFL learners serve as an ideal learner group for such a study concerning the acquisition of the singular *they*. This is because both the said internal (L1) and external sources of difficulty apply to Chinese speakers learning English. First, Mandarin is a pro-drop language and thus significantly different from English, a non-pro-drop language. A typical feature of the pronoun use of a pro-drop language is that the personal pronoun is frequently omitted and that nouns, instead of pronouns, are often used for reference maintenance (Hendriks, 2003). This means that while gender is often not encoded in Mandarin sentences, it usually is in English (see Antón-Méndez, 2010). Such a discrepancy between the two languages may make Mandarin-speaking learners more prone to making gender-related errors when using English. The origin of such errors, as noted by Antón-Méndez (2010), is thought to be located at the conceptual processing level since it involves “the composition of the preverbal message that guides grammatical encoding during language production” (p. 119). In an English pronoun-eliciting task, Antón-Méndez (2010) found that Spanish L2 speakers whose native language is a pro-drop language made significantly more gender errors for third-person singular nominative pronouns than French L2 speakers whose native language is a non-pro-drop language (4.30% vs. 68%, respectively). Antón-Méndez suggested that such an error pattern of pronoun use by Spanish L2 speakers could not have resulted from L1 transfer. The reason is that Spanish L2 speakers would have otherwise made more errors of nominative pronoun omission and that it is a failure of encoding gender information in the preverbal message (as described by Levelt, 1989) when producing English (see Dong et al., 2014). However, for Mandarin-speaking English learners, it is indeed possible that pronoun errors could result from L1 transfer (see Guo and Yuan, 2020). While Spanish has two equivalent third-person pronouns for the English *he* and *she*, these pronouns in Mandarin are homophones; that is, they have different spellings with the same pronunciation [*tā*]. Because of the influence from their native language, Chinese EFL speakers are often found to make gender-related errors in their use of pronouns in spoken English, such as *he* for *she* or *she* for *he* (Dong and Jia, 2011). Dong et al. (2014) conducted two self-paced reading experiments in an attempt to examine the underlying cause and mechanism of L1 transfer in the mishandling of English third-person pronouns of Chinese EFL learners. It was found that when highlighting the gender information of an antecedent with a human picture, Chinese EFL learners were particularly sensitive to the mismatching effect. In other words, the reading time for the pronoun that mismatched its antecedent in gender was longer than that for the pronoun that matched. The mismatching effect then disappeared when the human picture was removed. Based on these findings, Dong et al. (2014) concluded that Chinese EFL learners are not sensitive to the gender information encoded in the antecedent and that L1 transfer errors corresponding to English pronouns are a result of deficient processing of gender information in the conceptualizer.

An interesting question then arises as to whether similar difficulties relating to gender errors also occur in the acquisition of the singular *they* by Chinese EFL learners, and, if so, which account or mechanism explains such difficulties: Is it the failure of encoding gender information in the preverbal message (Antón-Méndez, 2010) or the deficient processing of gender information in the conceptualizer (Dong et al., 2014)? Based on the features and usages of the singular *they*, as discussed above, it is assumed that to acquire its use, L2 learners need to unlearn the plural feature [−PLURAL]—a contrastive element that the plural form of *they* possesses [+PLURAL]—and, at the same time, learn its gender feature, namely, generic, gender-neutral, or epicene. If this is the case, Chinese EFL learners may not have much difficulty with the gender feature of the singular *they* given that their pronoun errors mainly lie in the binary gender feature, as documented by Antón-Méndez (2010) and Dong et al. (2014). It then appears that the real difficulty for Chinese EFL learners may be located in unlearning the plural feature of the singular *they* [−PLURAL]. Furthermore, as discussed above, the choice of the singular *they* by native English speakers is also a sociolinguistic and pragmatic issue, and its acquisition is likely to be affected by L1 sociocultural norms. It can thus be hypothesized that the acquisition by Chinese EFL learners of the singular *they* is problematic due to both internal and external sources of the difficulty. For the internal source of difficulty, Chinese EFL learners would need to restructure their knowledge of the plural *they*, which may have already been internalized in their mental lexicon. There is also the external source of difficulty: EFL classroom settings in China, as previously mentioned, do not provide learners with sufficient input or exposure to the singular *they* to enable them to restructure and develop a new robust representation of it in their internal grammar systems. Against this backdrop, the present study attempts to answer the following two sets of research questions:

1. Can Chinese EFL learners develop native-like performance in the acceptance of the singular *they*? Is their performance modulated by their level of English proficiency?
2. Will the use of the singular *they* incur processing costs during its real-time interpretation? To what extent is the real-time interpretation of the singular *they* modulated by English proficiency levels of learners?

## Current Study

Two experiments were conducted to answer the research questions. Experiment 1 consisted of an untimed acceptability judgment test (AJT) to examine the acceptability of the singular *they* by two Chinese EFL groups with different proficiency levels. Research has shown that AJT tasks can simultaneously tap into participants’ implicit and explicit knowledge of L2 linguistic features (Gutiérrez, 2013; Suzuki, 2017; Plonsky et al., 2019). Experiment 2 entailed a SPR designed to examine the real-time processing by Chinese EFL learners of the singular *they*. As outlined above, the problem with the acquisition of the singular *they* by Chinese EFL learners might lie in the restructuring of their existing knowledge base of its plural feature. By examining both offline judgment and online processing data, the present study was able to test this supposition and helped provide a more complete picture of the acquisition of the singular *they* by Chinese EFL learners.

To avoid a potential priming effect from Experiment 1, we first conducted Experiment 2. However, to make the reporting more logical, we first present the results of Experiment 1.

### Experiment 1: Acceptability Judgment Test

#### Participants

Three groups of participants took part in the AJT. The first two groups were Chinese EFL learners, and the third group comprised native English speakers. The Chinese EFL learners were all recruited from a key university in an eastern city in China. One group consisted of 30 English-major postgraduate students, while the other consisted of 30 non-English-major postgraduate students from a variety of disciplines including material engineering, aeronautics and astronautics, and electrical engineering.

Although these two groups of non-native speakers were similar in age (see Table 1), they differed in terms of their English learning experiences. While they all started learning English from Grade 3 in primary school and collectively had 9 years of English in an instructional setting before entering university, the two groups had received a rather different English education in university. As undergraduates, the English majors had full-time courses of English reading, listening, speaking, and writing. In addition, they attended supplementary year-long courses focusing on English for Academic Purposes in linguistics and applied linguistics as postgraduates. On the other hand, the non-English majors had full-time courses in their majors as undergraduates, but only two English for General Purposes (EGP) courses each week. As postgraduates, they had two EGP courses each week but for only one semester. Therefore, the English majors had a greater exposure to English and, in turn, to the use of the singular *they* than the non-English majors through different learning activities (such as those relating to all four skills of reading, listening, speaking, and writing) and English courses (including both English-skill-oriented courses and content-based English courses).

**TABLE 1:** Demographic information of the two Chinese English as a Foreign Language (EFL)-learner groups.

Participants (*N* = 60)	Mean age	Mean English proficiency score (Max. = 40)
English majors (*n* = 30)	23.4 (0.92)	32.30 (3.31)
Non-English majors (*n* = 30)	23.9 (0.90)	28.10 (4.00)
		

Before the experiments began in earnest, all the participants took an English proficiency test (see section “Materials” for details of the test). The results indicated that the English-major postgraduate students achieved significantly higher scores in the test [*t*(55.99) = 4.322, *p* < 0.001, *d* = 1.12; see Table 1]. By including these two groups of non-native speakers, we were able to examine whether the acceptance of the singular *they* in an offline and an online task would be influenced by the English proficiency levels of participants.

The native English speakers were recruited among undergraduate students at an American university. We sent each potential participant an email to notify them of the purpose of the study and the materials to be used. If they agreed to participate, we then sent them a link from which they could retrieve the questionnaire and begin the test. In total, 19 native English speakers were recruited. Written consent was obtained from all three groups for their data to be included in the write-up.

#### Materials

##### Background Questionnaire

This questionnaire (provided in Supplementary Material 1) consisted of questions concerning the demographics of participants, including their age, gender, length of English learning, and—for the Chinese EFL groups only— whether and how long they had stayed in an English-speaking country.

##### English Proficiency Test

Following the practice of Jensen et al. (2020), we adopted a 40-item subset of the Oxford Proficiency test (provided in Supplementary Material 2) to assess the English proficiency of participants. The format of the test was multiple-choice, where the participants had to select one out of three options to fill in a blank, as illustrated in the following example:

(2) He is very well known ___ the world.
  A. all in B. all over C. in all.

##### Acceptability Judgment Test

This test consisted of 60 English sentences (provided in Supplementary Material 3), a sample of which is as follows:

(3) a. A truck driver must take a break every 4 hours, even if ___ (*he/she/they*) may not be tired, because driving for long periods of time without a break is not dangerous.
b. A clerk should create value for the company, even if ___ (*he/she/they*) may enjoy the holiday, because traveling is relaxing.
c. An adult should have an understanding of politics, even if ___ (*he/she/they*) may not want to be a politician, because adults have a right to vote.
d. Anyone who wants to be a teacher must go to university, even if ___ (*he/she/they*) may just want to be a pre-school teacher, because there is not a lot to learn before being able to teach effectively.

These sentences were each a complex sentence involving a main clause and two dependent clauses. The main clause began with a masculine (3a), feminine (3b), or neutral common noun (3c) modified by an indefinite determiner (e.g., *a truck driver*, *a nurse*, or *a runner*) or an indefinite pronoun (e.g., *anybody*, *anyone*; 3d). The common noun or the indefinite pronoun was the subject of the sentence and the only intended referent of the pronoun in the second clause. The first dependent clause began with the subordinate conjunction *even if* followed by a blank where a third-person English pronoun (i.e., *he*, *she*, *they*) was supposed to be added. The verb in the second clause was unmarked for a number so that its form was identical regardless of the pronoun used. The second dependent clause, led by the subordinate conjunction *because*, “provided a justification for the opinion expressed in the first two clauses and was included as a buffer,” so that reading time recorded in Experiment 2 “for the crucial second clause would not be contaminated by a reader’s wrap-up processing at the end of each sentence” (Foertsch and Gernsbacher, 1997, p. 108).

All the sentences were borrowed from the study by Speyer and Schleef (2019), but some modifications were made concerning the common nouns used in the main clauses to ensure that all the nouns had “a clear stereotypical connotation for either feminine or masculine” for Mandarin readers (Speyer and Schleef, 2019, p. 798). A questionnaire was created and distributed to a group of 50 participants selected from the same participant pool, as outlined above. The participants were asked to rank 80 nouns (60 chosen from the common nouns used by Speyer and Schleef and 20 created by the first author) on a Likert scale, where 1 = *extremely female*, 3 = *neutral*, and 5 = *extremely male*. All the nouns with a mean rating score of 2.19 (*SD* = 0.33) were classified as stereotypically female, and all the nouns with a mean rating score of 4.22 (*SD* = 0.23) as stereotypically male. Neutral nouns were those with a mean rating score of 3.84 (*SD* = 0.26).

**FIGURE 1:**
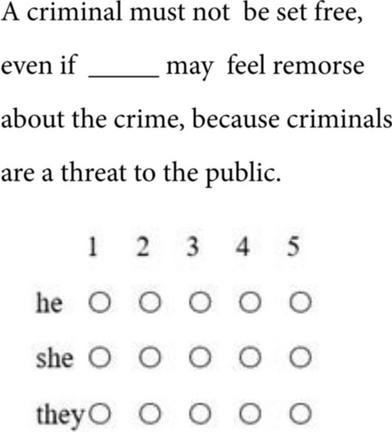
Example sentences displayed in the survey.

In the test, participants were asked to decide on the acceptability of each pronoun by filling in the blank on Likert 1–5 scales where 1 = *completely unacceptable* and 5 = *completely acceptable*. An example of the test is shown in Figure 1.

#### Procedure

The test for the Chinese EFL learners was conducted on the Chinese Survey Star platform, through which we sent all the participants an email and a link to the test. The participants first answered questions in the background questionnaire and then took the AJT, taking approximately 25–35 min to complete the entire task. The instructions were given to the participants in Mandarin both orally and in writing. They each received 20 RMB (equivalent to approximately 3 US dollars) for their participation.

The test for the native English speakers was run on Qualtrics, through which we sent the participants each an email and a link to the test. The participants first answered a set of questions concerning their background, including age, major, and language learning experience, and then took the AJT. The entire task took approximately 20 min. Upon completion, they each received an Amazon gift card of 10 USD.

#### Results

**FIGURE 2:**
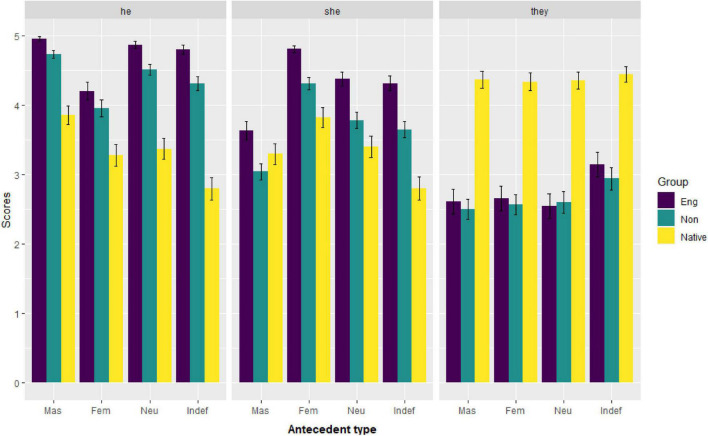
Mean acceptability of each pronoun as a function of the antecedent type by each group. (*Eng*, English majors; *Non*, non-English majors; *Native*, native English speakers; *Mas*, masculine; *Fem*, feminine; *Neu*, neutral; *Indef*, indefinite).

Figure 2 shows the mean acceptability score for each pronoun as a function of the antecedent type by the three groups of participants. It can be seen that, among the three groups of participants, native English speakers gave the lowest ratings to *he* and *she* in almost every type of antecedent, whereas they gave the highest ratings to the use of *they*. In marked contrast, the two groups of non-native speakers gave low ratings to the use of *they* in all types of antecedent but very high ratings to the use of both *he* and *she*.

All the data analyses in this study were performed using the statistical software R (R Development Core Team, 2020). To determine how the three groups of participants differed in their ratings of the use of the different pronouns among the different antecedent types, we fitted a generalized mixed-effects model with Poisson regression *via* the *glmer* function in the lme4 R package (Bates et al., 2015). There were three independent variables in the present study: *group*, *antecedent*, and *pronoun*. These variables were treated as the fixed-effects factors in the model, while the participants and the sentences they read were the random-effects factors in the model. We followed the “keep it maximal” rule proposed by Barr et al. (2013) when fitting the random-effects structure by including both by-subject and by-item random slopes and their intercepts for all the relevant fixed effects. We obtained *p*-values for the main effects and interactions of the three factors by using likelihood ratio tests *via* the *mixed ()* function in the *afex* package.

The results of the mixed-effects model indicated that both *pronoun* and *antecedent* had main effects [*pronoun*: χ^2^(2) = 500.99, *p* < 0.0001; *antecedent*: χ^2^(3) = 7.93, *p* = 0.047], but *group* had no main effect [χ^2^(2) = 3.35, *p* = 1.87]. No *antecedent* × *group* × *pronoun* interaction was found [χ^2^(12) = 16.00, *p* = 0.191], but a significant *group* × *pronoun* interaction was detected [χ^2^(4) = 1146.70, *p* < 0.001]. In addition, there were also a significant *group* × *antecedent* [χ^2^(6) = 79.39, *p* < 0.001] interaction and an *antecedent* × *pronoun* interaction [χ^2^(6) = 259.25, *p* < 0.001].

**FIGURE 3:**
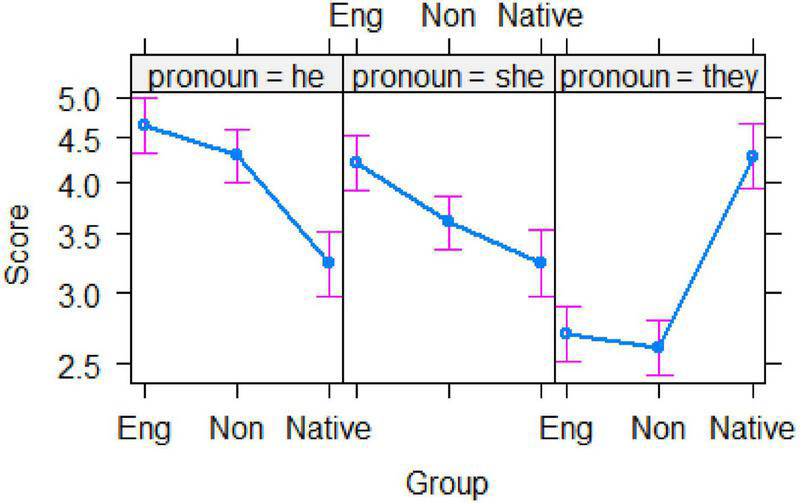
The interaction between *group* and *pronoun*. (*Eng*, English majors; *Non*, non-English majors; *Native*, native English speakers).

The effect of *group* × *pronoun* interaction can be clearly seen in Figure 3. Both groups of Chinese EFL learners gave significantly higher ratings to *he* than *she* (*z*s > 5.8, *p*s < 0.001), which were both significantly higher than their ratings to *they* (*z*s > 16.00, *p*s < 0.001). For the native English speakers, however, the rating of *he* was similar to that of *she* (*z* < 1.00, *p* > 0.90), but the ratings of these two pronouns were significantly lower than those of *they* (*z*s > 12.00, *p*s < 0.001). A notable difference was found in the ratings of the pronoun *they*. The ratings produced by both EFL groups were similar (*z* = 0.69, *p* = 0.92), but their ratings were much lower than those given by the native English speakers (*z*s > 8.0, *p*s < 0.001).

**FIGURE 4:**
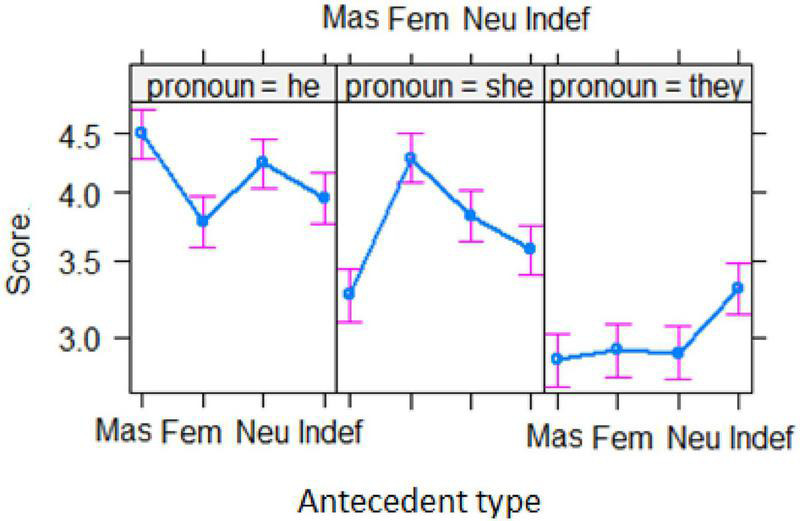
The interaction between *antecedent* and *pronoun*. (*Mas*, masculine; *Fem*, feminine; *Neu*, neutral; *Indef*, indefinite).

Figure 4 clearly displays the interaction effect between *antecedent* and *pronoun*. It was found that *he* was given the highest ratings in the masculine condition, higher than in any other types of conditions (*z*s > 3, *p*s < 0.001), and it was given the lowest ratings in the feminine condition, lower than both in masculine and neutral conditions (*z*s > 3, *p*s < 0.001). However, *he* was given similar ratings in the feminine and indefinite conditions (*z* = 0.899, *p* = 0.99). *She* received the highest ratings in the feminine condition, higher than in any other conditions (*z*s > 3, *p*s < 0.001) and received the lowest ratings in the masculine condition (*z*s > 3, *p*s < 0.001). In contrast, *they* was given the highest rating in the indefinite condition, higher than in any other three conditions (*z*s > 3, *p*s < 0.001) in which it received similar ratings.

**FIGURE 5:**
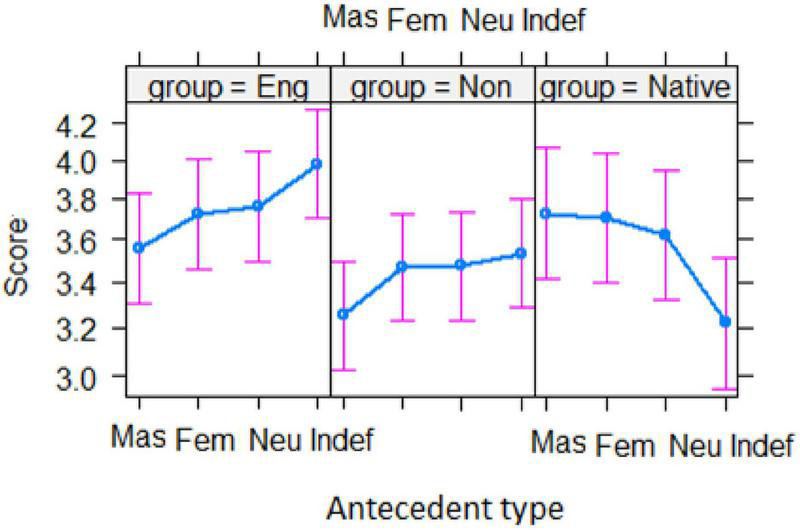
The interaction between *group* and *antecedent.* (*Eng*, English majors; *Non*, non-English majors; *Native*, native English speakers; *Mas*, masculine; *Fem*, feminine; *Neu*, neutral; *Indef*, indefinite).

Figure 5 displays the effect of *group* × *antecedent* interaction. As can be seen, the pattern of ratings of the two Chinese EFL groups was similar across the four types of antecedents as they gave the lowest ratings in the *masculine* condition but the highest ones in the *indefinite* condition. By contrast, the patterns of ratings by the native English speakers were the opposite, with the lowest ratings in the *indefinite* condition but the highest ratings in the *masculine* condition.

#### Discussion

The results from Experiment 1 demonstrated a notable difference in performance between the native English speakers and the two Chinese EFL groups. The native English speakers rated *they* as the most acceptable pronoun regardless of the antecedent type. Conversely, both groups of Chinese EFL learners rated *they* as the least acceptable among the three pronouns in any type of antecedent. Furthermore, they rated the singular masculine pronoun *he* as the most acceptable pronoun. As for whether their acceptance was modulated by their English proficiency levels, the results were negative since there was little difference in the performance between the two groups of Chinese EFL learners.

However, despite the difference from the native English speaker group, the two Chinese EFL groups did share some similarities with the former in their use of the three English third-person pronouns. First, the two singular pronouns *he* and *she* always received higher ratings in the stereotypical gender-matching antecedent type (e.g., *he* = *a police officer*) than in any other antecedent types (e.g., *she* = *a police officer*). This implies that Chinese EFL learners can develop a native-like representation of gender differences in the English pronoun system.

Another similarity between the two Chinese EFL groups and the native English speaker group is that *they* received the highest ratings in the *indefinite* condition compared to any other condition (as illustrated in Figure 2), although the singular *they* still received the lowest ratings among the three pronouns. This means that Chinese EFL learners would accept the use of the singular *they* in the *indefinite* condition where the gender and number are both unspecified, more so than in other antecedent types.

However, this experiment did not examine whether the dispreference of the singular *they* by Chinese EFL learners would lead to cognitive costs during their English reading comprehension and whether the L2 processing of the singular *they* would be affected by English proficiency levels of learners. To address these questions, Experiment 2 was conducted.

### Experiment 2: Self-Paced Reading Test

#### Participants

The same two groups of Chinese EFL learners who participated in Experiment 1 (English majors vs. non-English majors) took part in Experiment 2 except a week earlier. Given that these two experiments were different in design, the participants would unlikely have been influenced by their prior exposure to Experiment 2.^1^ They each received 20 RMB (equivalent to approximately 3 US dollars) for their participation in this experiment.

A group of 30 native English speakers was recruited to serve as the baseline for comparison; however, these participants did not take part in Experiment 1. The reason for recruiting a new group of native speakers is that while Experiment 1 could be completed *via* Qualtrics, Experiment 2 had to be conducted in a sound-proof booth *via* E-prime 2.0. Of these, 20 postgraduate students (aged between 22 and 31) were recruited from an American university, and the remaining 10 were recruited from Americans living in China. Four of them were graduate students from a key university in eastern China (aged between 22 and 23), and the other six were English teachers (aged between 32 and 38) to university students in China. They each received 20 USD for their participation.

#### Materials

The same 60 English sentences used in the AJT as introduced in Experiment 1 were also used in Experiment 2, albeit with one difference: the participants had to respond to a TRUE/FALSE question after each sentence. Examples of such questions are: *Does this sentence make sense?* and *Do you agree?* Answers to the questions were half yes (TRUE) and half no (FALSE), depending on the second dependent clauses of the sentences led by *because*. Instead of presenting the sentence segment by segment as per Foertsch and Gernsbacher (1997) and Speyer and Schleef (2019), each sentence in the current study was presented word by word, but each clause was displayed in three separate lines, as in the following example:

(4) A clerk/should/create/value/for/the/company/,/
even/if/*they/*may/enjoy/the/holiday/,/
because/traveling/is/relaxing/./

The reason that the experiment was conducted word by word was to reduce the possibility of the effect being washed out by the neighboring words when presented clause by clause (Marsden et al., 2018).

Three sets of materials were created to ensure that each sentence appeared with a different pronoun in each material set. The experiment was a 4 × 3 within-subjects design. The categories of *antecedent* and *pronoun* were both within-subjects variables, but the former was a between-items variable. Participants were randomly assigned one set of materials, and each sentence was presented in a random order in the testing session.

It should be noted that even though some sentences did not make logical sense, this should not have influenced the judgment in Experiment 1 and the reading response in Experiment 2. The reason is that, in both Experiments, decisions on the acceptability of the pronouns should have already been made before the participants read the last clause of each sentence, i.e., the part that affected the logicality of the sentence.

#### Procedure

The experiment was run in E-prime 2.0 *via* a moving-window technique (Schneider et al., 2002). Each sentence was presented in a word-by-word fashion from left to right, in black characters of 12-point typeface on a white background in the center of a 17-inch monitor. The participants completed the reading task individually in a private soundproof booth. Detailed oral instructions were given to each participant, but written instructions were also displayed on the computer screen before the test. Both these sets of instructions were administered in the L1 of participants. All the participants first read eight sentences as practice trials to familiarize themselves with the task. Feedback for their responses to the comprehension questions was provided in the practice trials, although no feedback was given in the actual test. Data from the practice trials were not included in the data analysis.

All participants were asked to stay focused and were encouraged to read at their own pace. The entire experiment lasted approximately 20–25 min. The presentation of each word in the sentences was initiated by the pressing of the space bar, and the word disappeared when the next appeared. This process ensured that the reader could not see the whole sentence on the screen.

#### Results

The native English speakers attained an accuracy of 91.89% in answering the questions following each sentence. The overall accuracy of the Chinese EFL learners was 86% (English majors: *M* = 88.3%, non-English majors: *M* = 83.9%). These results indicated that both L1 and the two EFL groups were focused and understood what they were reading during the experiment.

We analyzed the reading times of participants in three regions. Region 1, also *the critical region*, comprised the pronoun that served as the subject of the first dependent clause, namely, *they* in (4). Region 2 was *the post-critical region*, consisting of the word immediately following the critical region, namely, *may* in (4). Region 3, *the latter region*, incorporated the word immediately following the post-critical region, namely, *enjoy* in (4). The reading time (RT) scores of participants were first excluded for further analysis when they gave wrong answers to the TRUE/FALSE questions. Afterward, further data screening procedure for outliers was conducted. RT scores smaller than 150 ms or 2.5 standard deviations above the means were all excluded. As a result, 3.81% of the data were removed from the critical region, 5.40% of the data from the post-critical region, and 8.14% of the data from the latter region.

With the trimmed data, we fitted a mixed-effects model on the RT scores of participants in each region using the lme4 package (Bates et al., 2015). In the model, *group*, *antecedent*, and *pronoun* were the fixed-effects factors, while the participants and the words they read were random-effects factors. Following the same protocol as Experiment 1, we obtained *p*-values for the main effects and interactions of the fixed-effects factors by using likelihood ratio tests *via* the *mixed ()* function in the *afex* package.

**FIGURE 6:**
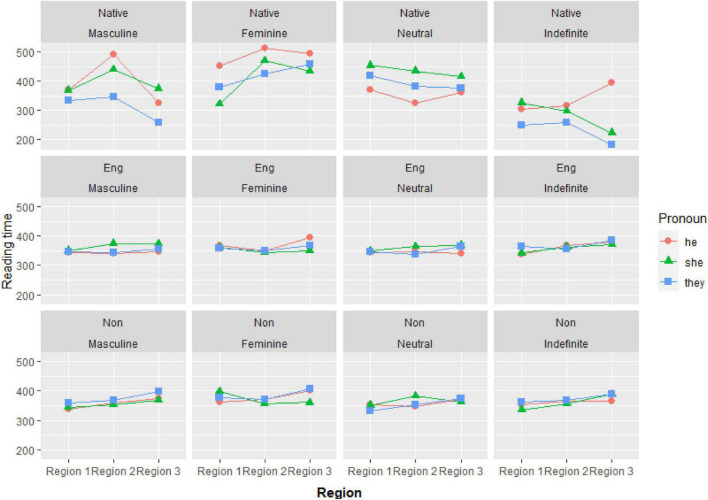
The mean reading times of each pronoun by each group as a function of the antecedent type in different regions. (*Native*, native English speakers; *Eng*, English majors; *Non*, non-English majors).

In addition, the preceding reading time was entered as a covariate (i.e., the time participants spent on reading the preceding region) to control the effect of the “temporal dependency” when modeling reaction times (see Baayen and Milin, 2010, p. 18). However, before entering the model, the scores of the preceding reading time were scaled and centered to overcome potential converging problems and to reduce the possibility of the multicollinearity of the model. For all the regions reported below, the absolute *t*-values associated with the coefficients of the preceding reading time were all fairly large (>4). This result showed that the inclusion of the preceding reading time in the model made it possible to reduce the problems of interdependence among the data points in the reading latencies and obtain a better estimation of the effects of the other fixed factors. Figure 6 displays the mean reading times of each pronoun by each group as a function of the antecedent type in different regions.

##### Region 1: The Critical Region

The results of the mixed-effects model indicated that neither *pronoun* [χ^2^(2) = 3.91, *p* = 0.141] nor *group* [χ^2^(2) = 1.05, *p* = 0.592] had main effects, but a significant main effect of *antecedent* was found [χ^2^(3) = 92.90, *p* < 0.001]. More importantly, there was a significant *group* × *pronoun* × *antecedent* interaction [χ^2^(12) = 116.88, *p* < 0.001].

*Post hoc* tests were subsequently conducted *via* the *emmeans ()* function in the *emmeans* package to isolate the effects of the three-way interaction. It was found that when the antecedent was masculine, native English speakers read the three pronouns at similar rates (*p*s > 0.50). For the feminine antecedent, they were the slowest in reading *he* and slower than *she* (β = 153.14, SE = 14.00, *z* = 10.95, *p* < 0.0001) and *they* (β = 100.56, SE = 14.40, *z* = 6.99, *p* < 0.0001), but no difference was found between *she* and *they* (β = 52.58, SE = 14.10, *z* = 3.73, *p* = 0.07).

When the antecedent was neutral, native English speakers were the fastest in reading *he*, faster than *she* (β = 74.47, SE = 15.00, *z* = 4.98, *p* < 0.001), but the differences between *he* and *they* or *she* and *they* were not significant (*p*s > 0.70). Finally, when the referent of the sentence was an indefinite pronoun, native English speakers read the three pronouns at similar rates. No significant differences were found in their reading times (*p*s > 0.18). For the two Chinese EFL groups, no significant differences were found in their reading times among the three pronouns in all the four antecedent types (*p*s > 0.89).

These results suggest that English native speakers were unaffected or even facilitated by the use of the singular *they* in their reading. Similarly, the two groups of Chinese EFL learners did not seem to be disrupted by the use of the singular *they*. However, as described by Juffs (1998, p. 127), it is possible that their response latencies might have “spilled over” onto the reading times of the following word or words.

##### Region 2: The Post-critical Region

The results indicated that *pronoun* [χ^2^(2) = 18.22, *p* < 0.001], *group* [χ^2^(2) = 7.90, *p* = 0.019], and *antecedent* [χ^2^(3) = 70.82, *p* < 0.001] all had main effects. A significant *group* × *pronoun* × *antecedent* interaction was found [χ^2^(12) = 58.02, *p* < 0.001].

*Post hoc* procedures revealed that when the antecedent was masculine, the native English speakers read *they* the fastest, faster than both *he* (β = 118.31, SE = 15.00, *z* = 7.91, *p* < 0.0001) and *she* (β = 63.95, SE = 14.60, *z* = 4.39, *p* = 0.006), but no significant difference was found between *he* and *she* (β = 54.36, SE = 15.10, *z* = 3.61, *p* = 0.11). For the feminine antecedent, the native English speakers were the slowest in reading *he*, slower than *they* (β = 78.77, SE = 15.40, *z* = 5.10, *p* = 0.0002), but no difference was found between *she* and *they* (β = 56.82, SE = 15.70, *z* = 3.62, *p* = 0.10).

When the antecedent was neutral, native English speakers read *he* the fastest, faster than *she* (β = 89.58, SE = 17.40, *z* = 5.16, *p* = 0.0001), but the differences between *he* and *they* or *she* and *they* were not significant (*p*s > 0.50). Finally, when the referent of the sentence was an indefinite pronoun, native English speakers read the three pronouns at similar rates (*p*s > 0.90).

For the two Chinese EFL groups, the English majors were faster in reading *they* than *she* (β = 20.60, SE = 7.41, *z* = 2.78, *p* = 0.015), and *he* than *she* (β = 36.60, SE = 15.20, *z* = 2.40, *p* < 0.0163), but no difference was found between *he* and *they* (*p* > 0.90). However, no other significant differences were found between any pronouns in the reading times for both the English-major and non-English-major groups in all the antecedent types (*p*s > 0.89).

##### Region 3: The Latter Region

The results indicated that both *pronoun* [χ^2^(2) = 15.59, *p* < 0.001] and *antecedent* [χ^2^(3) = 87.39, *p* < 0.001] had main effects, but *group* had no main effect [χ^2^(2) = 2.51, *p* = 0.019]. A significant *group* × *pronoun* × *antecedent* interaction emerged [χ^2^(12) = 108.85, *p* < 0.001].

*Post hoc* analyses indicated that when the antecedent was masculine, native English speakers read *they* the fastest, which was faster than both *she* (β = 99.01, SE = 15.00, *z* = 6.62, *p* < 0.0001) and *he* (β = 70.09, SE = 15.60, *z* = 4.48, *p* = 0.004). However, no significant difference was found between *they* and *he* (β = 28.92, SE = 15.40, *z* = 1.87, *p* = 0.99). For the feminine antecedent, they read the three pronouns at similar rates (*p*s > 0.18).

When the antecedent was neutral, as in Regions 1 and 2, native speakers read *he* the fastest, faster than *she* (β = −89.57, SE = 17.40, *z* = 5.16, *p* = 0.0001), but the differences between *he* and *they* and between *she* and *they* were not significant (*p*s > 0.50). Finally, in the indefinite antecedent, native English speakers read *they* the fastest, significantly faster than *he* (β = 204.57, SE = 16.80, *z* = 12.19, *p* < 0.0001) and she (β = 34.94, SE = 16.10, *z* = 2.17, *p* = 0.03).

For the two groups of Chinese EFL learners, it was found that the English majors, similar to Region 2, were significantly faster in reading *they* than *she* (β = 15.99, SE = 8.03, *z* = 2.00, *p* = 0.05). On the other hand, the non-English majors were significantly slower in reading *they* than *she* (β = −18.09, SE = 8.41, *z* = −2.15, *p* = 0.03). Overall, the non-English majors were slower than the English majors in reading *they* (β = −45.40, SE = 18.85, *z* = −2.41, *p* = 0.02).

#### Discussion

The results of Experiment 2 indicate that the reading of the native English speakers was not disrupted by the singular *they*. In some antecedent types, the pronoun *they* was read with greater facility than the other pronouns, whereas in other antecedent types, it was read with equal facility as the other pronouns (i.e., gender-matching pronouns). These results are in line with the findings reported by previous studies (Foertsch and Gernsbacher, 1997; Sanford and Filik, 2007; Vergoossen et al., 2020).

The results for the two Chinese EFL groups were much more complex. First, it appeared that the reading of the English majors, like that of the native English speakers, was not disrupted by the singular *they*. In all three regions and in all the antecedent types, there was no evidence that the English majors spent a longer time reading *they*. Instead, it was found that in both Regions 2 and 3, *they* was read with greater facility than *she*, a finding consistent with that of native English speakers. These results indicate that the singular *they* can serve as “a cognitively efficient substitute for generic he” for very advanced EFL learners, just as it can for native English speakers, as reported in Foertsch and Gernsbacher (1997, p. 106). For the non-English majors, however, a slightly different pattern of results was obtained. First, in most situations, the singular *they* did not seem to incur a longer reading time in that no additional cognitive processing was incurred, although it did in certain situations. For example, it was found that the non-English majors were consistently slower in reading *they* than *she*, regardless of the antecedent type. This would mean it would be less efficient for the non-English majors to substitute *they* for *she* in reading. In addition, it was found that the non-English majors were consistently slower in reading *they* than their English-major counterparts, further evidencing that the former did not develop the same level of acceptance for singular *they* as the latter did during online interpretations of L2 sentences.

Regarding the processing of the other two third-person singular pronouns (i.e., *he* vs. *she*), it should be noted that the non-English majors demonstrated major similarities with both their English-major counterparts and the native English speakers. Gender-mismatching pronouns incurred reliably longer reading times than gender-matching pronouns for all three groups (see Figure 6). This provides further support for the finding from Experiment 1 that both groups of EFL speakers were able to develop native-like gender representations in the acquisition of the English pronoun system, suggesting that gender sensitivity should not become an obstacle in the learning of L2 pronouns.

## General Discussion

In relation to the first set of research questions, we asked whether Chinese EFL learners would be able to develop native-like performance in the acceptance of the singular *they* and whether their performance would be modulated by their levels of English proficiency. Answering these points of enquiry hinges on the fact that Experiments 1 and 2 yielded an asymmetric pattern of results. First, in the AJT in Experiment 1, both Chinese EFL groups performed radically differently from the native English speaker group. While the native English speakers demonstrated a full acceptance of the singular *they* with all the antecedent types (as evidenced by the highest ratings given to *they*), both groups of Chinese EFL learners showed a reluctance to accept it in all the antecedent types (as evidenced by the lowest ratings given to *they*). Conversely, the SPR task used in Experiment 2 demonstrated that both Chinese EFL groups possessed great similarity to native English speakers in their real-time interpretation of the singular *they* during reading.

The second set of research questions enquired whether the use of the singular *they* would incur processing costs during its real-time interpretation and the extent to which the real-time interpretation of the singular *they* would be modulated by the English proficiency levels of the learners. Like the native English speakers, the higher-level English majors were not disrupted in their reading by the use of the singular *they* in all the antecedent types. For the lower-level non-English majors, there was no evidence to indicate that the singular *they* incurred longer reading times on most occasions (i.e., with different types of antecedents and in different regions). However, it was found that it would incur additional cognitive processing for *they* to be substituted for *she*, as indicated by the longer time that these participants took to read the singular *they*. Furthermore, the singular *they* did not seem as readily accessible for the non-English majors since it took a significantly longer time to process.

A likely reason for the performance asymmetry observed between the AJT and the SPR experiment by the L2 speakers appears to be the nature of the two different tasks and the features of the pronoun properties (i.e., gender and number). The central processes and the nature of knowledge and mechanism elicited by the two tasks were different (Marsden et al., 2018; Plonsky et al., 2019). It has been reported by researchers that untimed AJTs may lead to more use of explicit knowledge than timed tasks (Godfroid et al., 2015; Plonsky et al., 2019). In addition, several factors could help explain why both Chinese EFL groups did not find the singular *they* as acceptable in the untimed AJT. One source of difficulty for Chinese EFL learners to accept the singular *they* in the AJT might be that, historically, grammar—including the grammatical concept of singular vs. plural forms—has been the main focus of English language teaching and assessment, which is still largely true today (Xie, 2014; Wen, 2018). Grammatical rules, such as the rules for the use of English pronouns, are taught in an explicit and strict fashion, and constitute a major part of high-stakes English tests whether they be at the regional or national level. As a result, Chinese EFL learners are generally grammar conscious or sensitive when engaging in such tests, especially untimed AJTs. Another likely source of difficulty might be the fact that no pronoun counterpart of the English singular *they* can be found in Mandarin. A recent study by Wu, Li, and Qin (in press) indicated that the Mandarin plural pronoun *tamén* (counterpart of the English plural *they*) functioning as a singular pronoun is not acceptable for Mandarin native speakers. Such a discrepancy between the two languages has been found to be an important source of difficulty in L2 learning, particularly when it comes to the restructuring of existing knowledge for the plural feature of *they* [−PLURAL] and the adaptation to the sociocultural norms of the target language (Ellis, 2016; Shimanskaya and Slabakova, 2017). To overcome such a difficulty, Chinese EFL learners are likely to require positive evidence of the correct use of the singular *they* from their language input (Gabriele, 2009), yet such input is rare in Chinese classroom settings (Xie, 2014). L2 reading might compensate for this deficit, especially through extensive reading in English. However, the low saliency of the singular *they* may prevent learners from noticing it (Goldschneider and DeKeyser, 2001; Ellis, 2016).

In contrast to the untimed AJT task, in completing the online task in the SPR experiment, the focus of participants was not on grammaticality—including that of the use of pronouns—but on the meaning of the sentences they were reading. Compared with their behaviors in the AJT, both groups of L2 speakers seemed more tolerant of temporary ambiguity in this meaning-making process when encountering the singular *they*, which they rated as the least unacceptable in Experiment 1. This could be better explained in terms of pronoun resolution during L2 discourse comprehension (see Wu and Ma, 2020). According to the Mental Model Theory by Johnson-Laird and Garnham (1980), readers construct a mental model of the situations described by the words and expressions in question in the discourse. Take (2a) as an example: when encountering the pronoun, *they*, in the second clause, the reader must decide that *they* and *a truck driver* introduced in the first clause both indicate the same referent. What makes this possible and cognitively efficient is that all the materials used in the experiment are isolated sentences, and no other potential referent is available in the context. That is, *a truck driver* becomes the most salient or accessible referent available. This is consistent with the empirical evidence provided by other researchers. For example, it has been found that “…an ambiguous pronoun will prefer as its antecedent one that is most recently mentioned […] or one that appears in the subject position of the previous clause” (Roberts et al., 2008, p. 336). This provides the most probable account for the performance of the native English speakers. The fact that the English majors performed on a par with the native English speakers suggests that such L1 strategies were transferred to L2 reading (Wu, 2016). However, for the non-English majors, such transfer seemed incomplete given the fact that the singular *they* elicited a significantly longer reading time than the singular pronoun *she*, regardless of the antecedent type. However, it is unclear as to why a longer time was needed for *they* than for *she* but not for *they* than for *he*. The performance of this group in Experiment 1 may provide a possible explanation since it was found that the gendered singular pronoun received consistently higher ratings in the gender-matching antecedent type. For the non-English majors, referring to a female with a pronoun that was gender-neutral (and number-mismatching) might have been less socially acceptable and less probable than using a gender and number-matching pronoun (see Doherty and Conklin, 2017). Research has demonstrated that, with the improvement of the economic and social status of women in China, female address forms have also undergone important changes (Zhang, 2007), with forms showing discrimination having been replaced with those displaying respect and appreciation of equality. A case in point is the use of pronouns. Given that the singular *they* is not rated as acceptable (at least for the non-English majors), addressing a female using an ambiguous pronoun such as singular *they* may be seen as an act of disrespect or even arrogance. For EFL learners with a high level of English proficiency and a much greater exposure to English, the use of the singular *they* in such situations may be considered more acceptable, as demonstrated by the performance of the English majors. However, the reason that *they* did not elicit longer reading time than *he* for both groups of Chinese EFL learners may be that *he* has long been taught as a generic singular third-person pronoun to Chinese EFL learners (Dong et al., 2014). It may also be that male forms of address in China have remained more inclusive and tolerated than female forms of address, especially in the use of masculine pronouns.

However, native-like performance during online processing of the singular *they* did not mean a native-like mental representation of it in their grammar, as denoted by the performance of the two Chinese EFL groups on the AJT. A native-like mental representation of the singular *they* entails the development of the singular *they* as a distinct form in the mental lexicon (Paterson, 2014). If such is the goal of L2 learning, then even the upper-level English majors failed to reach it. Hence, concerted pedagogical efforts are needed to help Chinese EFL learners overcome the difficulty in acquiring the singular *they*.

The findings of the present study provide two important pedagogical implications. Firstly, an important source of difficulty in acquiring the singular *they* appears to lie in how knowledge of the singular *they* can be internalized in the mental lexicon of a learner. We believe that a possible solution is to provide sufficient positive evidence of the singular *they* in the input. This can be achieved through the provision of explicit information (EI). It has been reported that when EI is accompanied by task-essential practice, it can be effective in improving the accuracy and efficiency of L2 performance (McManus and Marsden, 2017; Lucas, 2020). Furthermore, because the singular *they* lacks saliency (for further discussion on salience, see Cintrón-Valentín and Ellis, 2016), it is important to make it more prominent in the input by explicitly raising awareness of it. In turn, this is likely to encourage its “noticing” (Benati, 2017). To facilitate this process, teachers can adopt input enhancement techniques, as suggested by Sharwood Smith (1991, 1993), such as italicizing or boldfacing particular features of a given text. According to the theory of Input Processing by VanPatten (2015), when learners attend to, or notice, input that necessitates comprehension *via* grammatical features, vital form-meaning connections are made. Accordingly, teachers can provide authentic texts that incorporate highlighted instances of the singular *they* in conjunction with pictures. This may also be helpful in restructuring existing knowledge of the plural *they* by reducing conceptual difficulty of L2 number learning, that is, “difficulties in mapping words to concepts” (Wagner et al., 2015, p. 1).

An issue closely related to this problem is the learning of the second important feature of the singular *they*, namely, the learning of its gender feature. Chinese EFL learners may not have much difficulty with the gender feature of the singular *they* given that their pronoun errors mainly lie in the binary gender feature. However, having little difficulty with these features does not mean actually learning them, as attested by the gender-information encoding account of Antón-Méndez (2010) or the L1 transfer account of Dong et al. (2014). Again, pictures provided when introducing the use of the singular *they* could facilitate the learning of both the number and gender features.

Secondly, it has been found that the difficulty in acquiring the singular *they* also involves the adaptation to the sociocultural norms of the target language. LaScotte (2021) has provided a suggestion for helping with this. “Teachers should address the reality that there are many who identify as neither *he* nor *she*” (p. 93). By doing so, teachers will help raise learner awareness of the existence of the singular *they* and its appropriate use. Moreover, teachers should help learners gain exposure to the singular *they* through reading English “newspapers, magazines, journals, grammars, and literary works,” where many examples for the singular *they* can be found (Mott-Smith, 2020, p. 1). Exposure to mass media and literary works will greatly help L2 learners in their adaptation of the target language’s sociocultural norms. It is pivotal to ensure that learners “understand the English language as actually used by native speakers as well as to promote respect for gender diversity in English language learners…” (Solomon, 2019, p. 69).

## Conclusion

We observed noticeable performance asymmetry between the AJT and the SPR experiments among the non-native speakers who participated in this study. The results of the two experiments seem to indicate that in the absence of a time constraint, the non-native speakers showed a heightened grammar sensitivity. Yet, with its presence, their grammatical sensitivity was reduced by a greater need to focus on meaning. We conclude that the difficulty for Chinese EFL learners to acquire singular *they* may lie in the restructuring of their existing knowledge of plural use of *they* [−PLURAL] in their mental lexicon and in the adaptation to the socio-cultural norms of the target language.

However, the generalizability of the findings is restricted due to methodological limitations. Firstly, the sample size of the native English speakers in Experiment 1 was small and involved only one age group, namely, undergraduate students. A larger sample size with different age groups might help gain a better understanding of how native and non-native speakers accept the singular *they* differently. In addition, only EFL learners of one L1 background (Mandarin) were examined. Future studies could profit from collecting data from learners of other L1 language backgrounds, especially those whose L1 pronoun systems and L1 sociocultural norms are different from both Mandarin and English. For example, L1 Danish speakers and L1 Arabic speakers may serve as ideal candidates due to the unique characteristics of these languages. In Danish, gender is structurally less complex, while in Arabic, speakers may have more influence from a “prevailing male-bias ideology” in L1 sociocultural norms (Stormbom, 2020, p.6). By doing so, researchers may gain a better understanding of the influence of cross-linguistic factors, such as L1 gender attributes and L1 sociocultural norms, in the learning of target features in general and pronoun systems in particular.

## Data Availability Statement

The original contributions presented in the study are included in the article/Supplementary Material, further inquiries can be directed to the corresponding author/s.

## Ethics Statement

The studies involving human participants were reviewed and approved by the Ethic Committee for Science and Technology of Shanghai Jiao Tong University. The patients/participants provided their written informed consent to participate in this study.

## Author Contributions

SW made the plan for the experiments, designed the materials, carried out the data analysis, and wrote the results and the discussion section. ZM wrote the literature review, the conclusion and prepared the references, and the Supplementary Material. SX helped to collected the data and the design of materials. All authors contributed to the article and approved the submitted version.

## Conflict of Interest

The authors declare that the research was conducted in the absence of any commercial or financial relationships that could be construed as a potential conflict of interest.

## Publisher’s Note

All claims expressed in this article are solely those of the authors and do not necessarily represent those of their affiliated organizations, or those of the publisher, the editors and the reviewers. Any product that may be evaluated in this article, or claim that may be made by its manufacturer, is not guaranteed or endorsed by the publisher.

## Footnotes

1 Our reasoning for using the same two groups of participants in the two experiments was that it would help us to infer their real-time reading processes by examining their offline data, thereby making it easier to integrate the findings of the two experiments. However, we do acknowledge that caution needs to be exercised in interpreting the results since the participants in Experiment 1 might have been influenced by their prior exposure to the reading in Experiment 2.
