# Supplementary material for: Acceptance and Online Interpretation of “Gender-Neutral Pronouns”: Performance Asymmetry by Chinese English as a Foreign Language Learners
Source: Front Psychol. 2022 Mar 2;12:765777. doi: 10.3389/fpsyg.2021.765777 (PMC8925987; doi:10.3389/fpsyg.2021.765777)
Supplement: Supplementary file 3 [file Data_Sheet_3.docx]

# SUPPLEMENTARY MATERIALS

# Part 1: Demographic survey

1. 您的性别 (Your gender):

男 (Male)

女 (Female)

1. 你的年级 (Your school level):

大学本科生 (College undergraduate students)

硕士研究生 (Postgraduate students)

3. 您的年龄是 (Your age):

4. 您正式学习英语的年数 (Your years of formal English study):

5. 您是否曾在英语国家生活/居住/旅游过一段时间？(Have you stayed, studied, or travelled in an English speaking country?)

6. 您在英语国家生活过多长时间？(How long have you lived in an English speaking country??)

# Part 2: English proficiency test

**The Standardized Oxford Proficiency test**

Part 1:

Instructions: Please complete the sentences by selecting the best answer from the available answers below.

1) Water ________ at a temperature of 100° C.

 A. is to boil

 B. is boiling

 C. boils

2) In some countries ________ very hot all the time.

 A. there is

 B. is

 C. it is

3) In cold countries people wear thick clothes _________ warm.

 A. for keeping

 B. to keep

 C. for to keep

4) In England people are always talking about _________.

 A. a weather

 B. the weather

 C. weather

5) In some places __________ almost every day.

 A. it rains

 B. there rains

 C. it raining

6) In deserts there isn’t _________ grass.

 A. the

 B. some

 C. any

7) Places near the Equator have ________ weather even in the cold season.

 A. a warm

 B. the warm

 C. warm

8) In England ____________ time of year is usually from December to February.

 A. coldest

 B. the coldest

 C. colder

9) ____________ people don’t know what it’s like in other countries.

 A. The most

 B. Most of

 C. Most

10) Very ________ people can travel abroad.

 A. less

 B. little

 C. few

11) Mohammed Ali ___________ his first world title fight in 1960.

 A. has won

 B. won

 C. is winning

12) After he ___________ an Olympic gold medal, he became a professional boxer.

 A. had won

 B. have won

 C. was winning

13) His religious beliefs _____________ change his name when he became a champion.

 A. have made him

 B. made him to

 C. made him

14) If he __________ lost his first fight with Sonny Liston, no one would have been

surprised.

 A. has

 B. would have

 C. had

15) He has traveled a lot ___________ as a boxer and as a world-famous personality.

 A. both

 B. and

 C. or

16) He is very well known _____________ the world.

 A. all in

 B. all over

 C. in all

17) Many people _______________ he was the greatest boxer of all time.

 A. is believing

 B. are believing

 C. believe

18) To be the best ___________ the world is not easy.

 A. from

 B. in

 C. of

19) Like any top sportsman, Ali ___________ train very hard.

 A. had to

 B. must

 C. should

20) Even though he has now lost his title, people _________ always remember him as a champion.

 A. would

 B. will

 C. did

Part 2:

Instructions: The following twenty questions form a continuous story. Please complete the sentences by selecting the best answer from the available answers below.

21) The history of _________________ is

 A. airplane

 B. the airplane

 C. an airplane

22) _____________ short one. For many centuries men

 A. quite a

 B. a quite

 C. quite

23) _________________ to fly, but with

 A. are trying

 B. try

 C. had tried

24) ______________ success. In the 19th century a few people

 A. little

 B. few

 C. a little

25) succeeded _________________ in balloons. But it wasn’t until

 A. to fly

 B. in flying

 C. into flying

26) the beginning of ________________ century that anybody

 A. last

 B. next

 C. that

27) __________ able to fly in a machine

 A. were

 B. is

 C. was

28) ________________ was heavier than air, in other words, in

 A. who

 B. which

 C. what

29) _______________ we now call a ‘plane’. The first people to achieve

 A. who

 B. which

 C. what

30) ‘powered flight’ were the Wright brothers. __________ was the machine

 A. His

 B. Their

 C. Theirs

31) which was the forerunner of the Jumbo jets and supersonic airliners that are

___________ common

 A. such

 B. such a

 C. some

32) sight today. They ________________ hardly have imagined that in 1969,

 A. could

 B. should

 C. couldn’t

33) ____________________ more than half a century later,

 A. not much

 B. not many

 C. no much

34) a man ___________________ landed on the moon.

 A. will be

 B. had been

 C. would have

35) Already __________ is taking the first steps towards the stars.

 A. a man

 B. man

 C. the man

36) Although space satellites have existed ____________ less

 A. since

 B. during

 C. for

37) than forty years, we are now dependent __________ them for all

 A. from

 B. of

 C. on

38) kinds of __________________. Not only

 A. informations

 B. information

 C. an information

39) ________________ being used for scientific research in

 A. are they

 B. they are

 C. there are

40) space, but also to see what kind of weather ________________.

 A. is coming

 B. comes

 C. coming

# Part 3: Acceptability judgment test questions for Experiment1/Self-paced reading experiment materials for Experiment 2

1. A criminal must not be set free, even if _____ may feel remorse about the crime, because criminals are a threat to the public.

1 2 3 4 5

he ○ ○ ○ ○ ○

she ○ ○ ○ ○ ○

they ○ ○ ○ ○ ○

2. Anyone who wants to be a teacher must go to university, even if _____ may just want to be a pre-school teacher, because there is not a lot to learn before being able to teach effectively.

1 2 3 4 5

he ○ ○ ○ ○ ○

she ○ ○ ○ ○ ○

they ○ ○ ○ ○ ○

3. A carpenter must know about different types of wood, even if _____ may think it is not important, because wood looks funny.

1 2 3 4 5

he ○ ○ ○ ○ ○

she ○ ○ ○ ○ ○

they ○ ○ ○ ○ ○

4. A clerk should create value for the company, even if _____ may enjoy the holiday, because travelling is relaxing.

1 2 3 4 5

he ○ ○ ○ ○ ○

she ○ ○ ○ ○ ○

they ○ ○ ○ ○ ○

5. An elderly person can still ride a bike, even if _____ may already be over 90, because being old doesn't necessarily mean that one can't ride.

1 2 3 4 5

he ○ ○ ○ ○ ○

she ○ ○ ○ ○ ○

they ○ ○ ○ ○ ○

6. A pensioner (退休人员) should be allowed to work, even if _____ may not need the money, because some pensioners like shouting.

1 2 3 4 5

he ○ ○ ○ ○ ○

she ○ ○ ○ ○ ○

they ○ ○ ○ ○ ○

7. An Austrian doesn't have to wear traditional costumes all the time, even if _____ may believe that it looks great, because on most days Austrians wear normal clothes.

1 2 3 4 5

he ○ ○ ○ ○ ○

she ○ ○ ○ ○ ○

they ○ ○ ○ ○ ○

8. A journalist has to report the truth, even if _____ may fear that some people won't like it, because it’s a journalist's duty to inform the public accurately.

1 2 3 4 5

he ○ ○ ○ ○ ○

she ○ ○ ○ ○ ○

they ○ ○ ○ ○ ○

9. Someone must feed the cat, even if _____ might not like the cat, because the cat will die if no one feeds it.

1 2 3 4 5

he ○ ○ ○ ○ ○

she ○ ○ ○ ○ ○

they ○ ○ ○ ○ ○

10. An athlete must know skills to win, even if _____ may not win every time, because having an ambition is human nature.

1 2 3 4 5

he ○ ○ ○ ○ ○

she ○ ○ ○ ○ ○

they ○ ○ ○ ○ ○

11. Everyone should use resources responsibly, even if _____ may not believe in man-made global warming, because our resources are finite.

1 2 3 4 5

he ○ ○ ○ ○ ○

she ○ ○ ○ ○ ○

they ○ ○ ○ ○ ○

12. An artist should have an additional job, even if _____ may be a talented artist, because being an artist doesn't provide one with a regular income all the time.

1 2 3 4 5

he ○ ○ ○ ○ ○

she ○ ○ ○ ○ ○

they ○ ○ ○ ○ ○

13. A pre-school teacher has to remain calm at all times, even if _____ may be stressed, because stress affects children positively.

1 2 3 4 5

he ○ ○ ○ ○ ○

she ○ ○ ○ ○ ○

they ○ ○ ○ ○ ○

14. Everyone should look after the environment, even if _____ may not believe in global warming, because excessive human activities may damage the environment.

1 2 3 4 5

he ○ ○ ○ ○ ○

she ○ ○ ○ ○ ○

they ○ ○ ○ ○ ○

15. An adult should have an understanding of politics, even if _____ may not want to be a politician, because vote doesn't need political knowledge.

1 2 3 4 5

he ○ ○ ○ ○ ○

she ○ ○ ○ ○ ○

they ○ ○ ○ ○ ○

16. A sailor must know how to swim, even if _____ may not know how to steer the ship, because it is not easy to be a helm (舵手).

1 2 3 4 5

he ○ ○ ○ ○ ○

she ○ ○ ○ ○ ○

they ○ ○ ○ ○ ○

17. A construction worker may not drink alcohol when working, even if _____ may have nearly finished the work for the day, because working while drunk is very dangerous.

1 2 3 4 5

he ○ ○ ○ ○ ○

she ○ ○ ○ ○ ○

they ○ ○ ○ ○ ○

18. A fire fighter must be very careful, even if _____ may be on a training mission, because otherwise it will be dangerous during rescues.

1 2 3 4 5

he ○ ○ ○ ○ ○

she ○ ○ ○ ○ ○

they ○ ○ ○ ○ ○

19. A car mechanic must know a lot about various types of cars, even if _____ may usually repair the same type of car, because knowledge is completely useless.

1 2 3 4 5

he ○ ○ ○ ○ ○

she ○ ○ ○ ○ ○

they ○ ○ ○ ○ ○

20. Anyone can learn how to play an instrument, even if _____ may not have the best sense of rhythm, because watching TV can be trained.

1 2 3 4 5

he ○ ○ ○ ○ ○

she ○ ○ ○ ○ ○

they ○ ○ ○ ○ ○

要求同上页

21. A housekeeper has to clean the toilet, even if _____ may find it disgusting, because that's the housekeeper's duty.

1 2 3 4 5

he ○ ○ ○ ○ ○

she ○ ○ ○ ○ ○

they ○ ○ ○ ○ ○

22. An electrician （电工） must be careful at all times, even if _____ may be just performing a routine job, because handling electricity is not dangerous.

1 2 3 4 5

he ○ ○ ○ ○ ○

she ○ ○ ○ ○ ○

they ○ ○ ○ ○ ○

23. A kid can learn a lot, even if _____ may not have the highest IQ, because the ability to learn is useless.

1 2 3 4 5

he ○ ○ ○ ○ ○

she ○ ○ ○ ○ ○

they ○ ○ ○ ○ ○

24. A farmer must be able to deal with planting, even if _____ may find it hard, because growing plants is one of a farmer's major jobs.

1 2 3 4 5

he ○ ○ ○ ○ ○

she ○ ○ ○ ○ ○

they ○ ○ ○ ○ ○

25. Somebody must have taken the key, even if _____ may not have intended to take it, because it can be found everywhere.

1 2 3 4 5

he ○ ○ ○ ○ ○

she ○ ○ ○ ○ ○

they ○ ○ ○ ○ ○

26. A librarian must know how to read, even if _____ may not read many books, because readers are nice.

1 2 3 4 5

he ○ ○ ○ ○ ○

she ○ ○ ○ ○ ○

they ○ ○ ○ ○ ○

27. A pilot must not fly when tired, even if _____ may delay the flight by not flying, because singing is fascinating.

1 2 3 4 5

he ○ ○ ○ ○ ○

she ○ ○ ○ ○ ○

they ○ ○ ○ ○ ○

28. A nanny (保姆) should not be working when sick, even if _____ may want to, because the nanny could not go abroad otherwise.

1 2 3 4 5

he ○ ○ ○ ○ ○

she ○ ○ ○ ○ ○

they ○ ○ ○ ○ ○

29. Everyone must hand in the exam now, even if _____ may not have finished it, because the swimming pool is cold.

1 2 3 4 5

he ○ ○ ○ ○ ○

she ○ ○ ○ ○ ○

they ○ ○ ○ ○ ○

30. A hairdresser cannot just cut a customer's hair very short, even if _____ may think that it would suit the customer, because it’s the customer's decision.

1 2 3 4 5

he ○ ○ ○ ○ ○

she ○ ○ ○ ○ ○

they ○ ○ ○ ○ ○

31. A child must follow teachers' instructions, even if _____ may not want to, because parents don't like children.

1 2 3 4 5

he ○ ○ ○ ○ ○

she ○ ○ ○ ○ ○

they ○ ○ ○ ○ ○

32. A model has to be in good shape, even if _____ may not want to be a fitness model, because lots of fish are swimming in the sea.

1 2 3 4 5

he ○ ○ ○ ○ ○

she ○ ○ ○ ○ ○

they ○ ○ ○ ○ ○

33. A secondary-school teacher must have a good general education, even if _____ may only teach, because pupils may ask the teacher questions.

1 2 3 4 5

he ○ ○ ○ ○ ○

she ○ ○ ○ ○ ○

they ○ ○ ○ ○ ○

34. A childcare worker should know how to draw, even if _____ may not be good at drawing, because children love drawing.

1 2 3 4 5

he ○ ○ ○ ○ ○

she ○ ○ ○ ○ ○

they ○ ○ ○ ○ ○

35. Anyone can be successful, even if _____ may not have the best education, because anything is possible if one works hard enough.

1 2 3 4 5

he ○ ○ ○ ○ ○

she ○ ○ ○ ○ ○

they ○ ○ ○ ○ ○

36. A baby-sitter must not look after baby when sick, even if _____ may receive only reduced pay, because the baby-sitter could infect babies.

1 2 3 4 5

he ○ ○ ○ ○ ○

she ○ ○ ○ ○ ○

they ○ ○ ○ ○ ○

37. A midwife must feel comfortable to see blood, even if _____ may mainly work in the after care of a baby, because midwifes don't like skiing.

1 2 3 4 5

he ○ ○ ○ ○ ○

she ○ ○ ○ ○ ○

they ○ ○ ○ ○ ○

38. Someone must empty the bin now, even if _____ may have emptied it yesterday, because it is empty again.

1 2 3 4 5

he ○ ○ ○ ○ ○

she ○ ○ ○ ○ ○

they ○ ○ ○ ○ ○

39. A guide must know a lot about history, even if _____ may only need to tell stories of some places of interest, because some tourists may ask more about history.

1 2 3 4 5

he ○ ○ ○ ○ ○

she ○ ○ ○ ○ ○

they ○ ○ ○ ○ ○

40. Anybody who wants to get a good grade must study hard, even if _____ might be happy with an average grade, because the exam is just for fun.

1 2 3 4 5

he ○ ○ ○ ○ ○

she ○ ○ ○ ○ ○

they ○ ○ ○ ○ ○

41. Anyone should be able to go to school, even if _____ may live in a remote rural area, because going to school is a waste of time.

1 2 3 4 5

he ○ ○ ○ ○ ○

she ○ ○ ○ ○ ○

they ○ ○ ○ ○ ○

42. A secretary must have good computer skills, even if _____ may only need type on a computer, because fast typing doesn't require typing skills.

1 2 3 4 5

he ○ ○ ○ ○ ○

she ○ ○ ○ ○ ○

they ○ ○ ○ ○ ○

43. A day care worker must be very understanding, even if _____ may be annoyed by a child, because children cannot always control their behaviour.

1 2 3 4 5

he ○ ○ ○ ○ ○

she ○ ○ ○ ○ ○

they ○ ○ ○ ○ ○

44. A translator has to know quite a lot about the target language, even if _____ may speak the language perfectly, because it is not easy to translate different languages.

1 2 3 4 5

he ○ ○ ○ ○ ○

she ○ ○ ○ ○ ○

they ○ ○ ○ ○ ○

45. Anybody can learn how to be a handstand (倒立), even if _____ may not be very fit for this role, because being a handstand only requires regular practice.

1 2 3 4 5

he ○ ○ ○ ○ ○

she ○ ○ ○ ○ ○

they ○ ○ ○ ○ ○

46. An astronaut must be able to concentrate when tired, even if _____ may find it not easy to realize, because flying into outer space doesn't require working very long hours.

1 2 3 4 5

he ○ ○ ○ ○ ○

she ○ ○ ○ ○ ○

they ○ ○ ○ ○ ○

47. A student must work very hard, even if _____ may not want to be the best, because a student needs to pass course exams.

1 2 3 4 5

he ○ ○ ○ ○ ○

she ○ ○ ○ ○ ○

they ○ ○ ○ ○ ○

48. A computer programmer should have studied computer science, even if _____ may already know a lot about programming, because a scientist is nice.

1 2 3 4 5

he ○ ○ ○ ○ ○

she ○ ○ ○ ○ ○

they ○ ○ ○ ○ ○

49. A police officer has many responsibilities, even if _____ may not be in a very high position, because being a police officer is the funniest job in the world.

1 2 3 4 5

he ○ ○ ○ ○ ○

she ○ ○ ○ ○ ○

they ○ ○ ○ ○ ○

50. Everyone can complete this assignment on time, even if _____ may have many other things to do, because this assignment is very easy.

1 2 3 4 5

he ○ ○ ○ ○ ○

she ○ ○ ○ ○ ○

they ○ ○ ○ ○ ○

51. One must tell the truth, even if _____ may hurt someone's feelings, because telling the truth is usually the right choice.

1 2 3 4 5

he ○ ○ ○ ○ ○

she ○ ○ ○ ○ ○

they ○ ○ ○ ○ ○

52. A teenager can be very annoying, even if _____ may not behave annoyingly on purpose, because a teenager's hormones (荷尔蒙) can cause mood swings.

1 2 3 4 5

he ○ ○ ○ ○ ○

she ○ ○ ○ ○ ○

they ○ ○ ○ ○ ○

53. A military officer must be able to handle a gun correctly, even if _____ may not want to use it, because the officer might encounter a situation where using a gun is necessary.

1 2 3 4 5

he ○ ○ ○ ○ ○

she ○ ○ ○ ○ ○

they ○ ○ ○ ○ ○

54. A pharmacist (药剂师) must know how drugs work, even if _____ may not prescribe drugs, because a pharmacist has to explain patients how to use drugs.

1 2 3 4 5

he ○ ○ ○ ○ ○

she ○ ○ ○ ○ ○

they ○ ○ ○ ○ ○

55. Anybody can enjoy sports, even if _____ may not be very fit, because enjoying sports only requires eating a lot.

1 2 3 4 5

he ○ ○ ○ ○ ○

she ○ ○ ○ ○ ○

they ○ ○ ○ ○ ○

56. A mechanic must know a lot about machines, even if _____ may only work with other machinery, because lots of machines are very beautiful.

1 2 3 4 5

he ○ ○ ○ ○ ○

she ○ ○ ○ ○ ○

they ○ ○ ○ ○ ○

57. A researcher should not hate teaching, even if _____ may be primarily interested in doing research, because a large part of the researcher's job consists of teaching students.

1 2 3 4 5

he ○ ○ ○ ○ ○

she ○ ○ ○ ○ ○

they ○ ○ ○ ○ ○

58. A truck driver must take a break every four hours, even if _____ may not be tired, because driving for long periods of time without a break is not dangerous.

1 2 3 4 5

he ○ ○ ○ ○ ○

she ○ ○ ○ ○ ○

they ○ ○ ○ ○ ○

59. A primary school teacher must like music, even if _____ may not be a great musician, because children can learn nothing from music.

1 2 3 4 5

he ○ ○ ○ ○ ○

she ○ ○ ○ ○ ○

they ○ ○ ○ ○ ○

60. A kindergarten teacher must know developmental psychology, even if _____ may not be interested in it, because working with children doesn't require any skills.

1 2 3 4 5

he ○ ○ ○ ○ ○

she ○ ○ ○ ○ ○

they ○ ○ ○ ○ ○
